# Predicting 30-day mortality using point-of-care testing; an external validation and derivation study

**DOI:** 10.1371/journal.pone.0239318

**Published:** 2020-09-24

**Authors:** Anniek Brink, Romy Schuttevaer, Jelmer Alsma, Robert Zietse, Stephanie Catherine Elisabeth Schuit, Hester Floor Lingsma

**Affiliations:** 1 Department of Internal Medicine, Section Acute Medicine, Erasmus MC University Medical Center Rotterdam, Rotterdam, the Netherlands; 2 Department of Public Health, Erasmus University Medical Center, Rotterdam, the Netherlands; Technion - Israel Institute of Technology, ISRAEL

## Abstract

**Background:**

Early risk stratification for guiding treatment priority in the emergency department (ED) is becoming increasingly important. Existing prediction models typically use demographics, vital signs and laboratory parameters. Laboratory-based models require blood testing, which may cause substantial delay. However, these delays can be prevented by the use of point-of-care testing (POCT), where results are readily available. We aimed to externally validate a laboratory-based model for mortality and subsequently assessed whether a POCT model yields comparable performance.

**Methods:**

All adult patients visiting the ED of a university hospital between January 1^st^, 2012 and December 31^st^, 2016 were retrospectively reviewed for inclusion. Primary outcome was defined as 30-day mortality after ED presentation. We externally validated one existing prediction model including age, glucose, urea, sodium, haemoglobin, platelet count and white blood cell count. We assessed the predictive performance by discrimination, expressed as Area under the Curve (AUC). We compared the existing model to an equivalent model using predictors that are available with POCT (i.e. glucose, urea, sodium and haemoglobin). Additionally, we internally validated these models with bootstrapping.

**Results:**

We included 34,437 patients of whom 1,942 (5.6%) died within 30 days. The AUC of the laboratory-based model was 0.794. We refitted this model to our ED population and found an AUC of 0.812, which decreased only slightly to 0.790 with only POCT parameters.

**Conclusions:**

Our POCT-model performs similar to existing laboratory-based models in identifying patients at high risk for mortality, with results available within minutes. Although the model needs further validation and evaluation, it shows the potential of POCT for early risk stratification in the ED.

## Introduction

Identifying patients in the Emergency Department (ED) at risk of dying remains challenging. The existing prediction models are typically based on demographics and vital signs.

Triage systems are initially used to identify the most severely ill patients. However, current triage systems, such as the Emergency Severity Index (ESI) [1] and the Manchester Triage System (MTS) [2], were mainly introduced for trauma patients. The performance of triage systems in all ED patients is poor [3–5]. Early warning scores (EWS) are also used in the ED, either as replacement or as addition to triage [6, 7]. Examples of EWS are the Modified Early Warning Score (MEWS) and the National Early Warning Score (NEWS). However, EWS are developed to detect inpatient clinical deterioration and the predictive value of EWS for mortality in the ED varies [8, 9] and both validation and calibration are inadequate or lacking [10].

Using laboratory parameters is another approach for early risk stratification. An advantage of laboratory parameters in prediction models is their objective measurement. Asadollahi et al. provided a laboratory-based prediction model derived from 1,650 acute medical and surgical patients, which performed well with an AUC of 0.848 [11]. The model was internally-externally validated using data from the same hospital in a different period of time. The model uses age, glucose, urea, sodium, haemoglobin, platelets and white blood cell count as predictors. These six laboratory parameters were selected from a large array of potential parameters and are known to correlate with adverse outcome [12–15]. Laboratory models require blood testing and can therefore cause a substantial delay if blood samples are analysed by a central laboratory. However, these delays might be prevented by point-of-care testing (POCT), which yields results within minutes [16, 17].

The aim of this study is to determine whether a laboratory model can be implemented using only POCT laboratory testing. Since external validation is a critical step to implementation in clinical practice, and to potentially improve the feasibility of the model, the first aim of this study is to externally validate the laboratory-based model by Asadollahi et al. [11] in a large unselected population of ED patients. The second aim is to assess whether a model based only on POCT available laboratory parameters yields comparable performance.

## Methods

### Study design and setting

We performed a retrospective cohort study in the ED of the Erasmus University Medical Center Rotterdam (Erasmus MC), Rotterdam, the Netherlands, which is a large tertiary referral centre, situated in an urban area. The ED has approximately 32,000 visits annually. Data from all patients were automatically extracted from the electronic health records on a regular basis and collected in a database.

### Selection of participants

We included all ED visits from January 1^st^ 2012 up to December 31^st^ 2016. Adult patients, aged 18 years and over, in which laboratory diagnostics were performed were selected for this study. Per patient only the first visit to the ED was included.

### Measurements and outcomes

We extracted demographic data (i.e. sex, age) and presenting vital parameters (i.e. body temperature, heart rate, respiratory rate, oxygen saturation, blood pressure and consciousness level using AVPU-scale). Furthermore, we extracted acuity scale according to MTS category, disposition (i.e. in hospital admission), and arrival (i.e. by ambulance). In line with the study of Asadollahi et al. we extracted haemoglobin, serum sodium, plasma glucose, white blood cell count, serum urea and platelet count. These laboratory values were afterwards categorized (Table 1).

**Table 1:** Laboratory tests with its cut-off points.

Parameter (Unit)	Reference range
Age≥65 (years)	
Urea >7.0 (mmol/L)	2.5–7.5 (mmol/L)
Haemoglobin <12.0 (g/dL) (7.45 mmol/L)	♂: 14–17.5 (g/dL)
	♀: 12.3–15.3 (g/dL)
Sodium <135 (mmol/L)	135–145 (mmol/L)
Glucose >7.0 (mmol/L)	<7.8 (mmol/L)
White blood count >10.0 (*10^9^/L)	4.0–10.0 (*10^9^/L)
Platelet count <150 (*10^9^/L)	150–400 (*10^9^/L)

Haemoglobin levels were converted from mmol/L to g/dL [11]. Subsequently, we selected the laboratory parameters that are measurable with POCT, i.e. glucose, urea, sodium and haemoglobin. At Erasmus MC, the ABL800 FLEX (Radiometer America Inc., Westlake, OH) blood gas analyser for POCT is used, which yields results within two minutes. Outcome was defined as 30-day mortality after the index ED visit. Mortality data were retrieved from the patient records, which are linked to municipal mortality records. The Medical Ethics Committee of the Erasmus MC reviewed the study and concluded that our study did not fall under the scope of the Medical Research Involving Human Subjects Act and therefore no informed consent needed to be obtained.

### Statistical analysis

Patient characteristics were presented as mean (SD), median (interquartile range (IQR)) or absolute numbers (percentage), when appropriate. Missing data were handled using multiple imputations (n = 5) with a chained equations procedure, which means that the expected value of the missing data point is estimated based on the available data.

We examined all patient characteristics of patients that were alive versus the patients who died within 30 days from the index ED visit. Data were compared using Pearson chi-squared tests or unpaired t-tests, based on distribution of data.

Model performance of the laboratory-based model was described as discrimination and calibration. Discrimination was assessed using the area under the Receiver Operating Characteristic-curve (AUC). We assessed calibration with a calibration plot in which the slope indicates the relation between the observed and the predicted outcome (i.e. ideally close to 1) and the intercept indicates whether the predictions are systematically deviant (i.e. ideally close to 0). We calculated likelihood ratios for all cut-off points from the total score and determined the ideal cut-off point using Youden’s index (i.e. the cut-off point combining the optimal sensitivity and specificity). Interval likelihood ratios were established for several different intervals. Furthermore, we refitted the model on our data and subsequently reduced the model by only including parameters which could be tested using POCT. These models were internally validated using five hundred times bootstrap resampling.

Analyses were conducted with IBM SPSS Statistics for Windows version 26 (IBM Corp., Armonk, New York, USA) and R statistics version 3.6.1. A significance level of p<0.05 was considered as statistically significant.

## Results

### Patient characteristics

**Fig 1:**
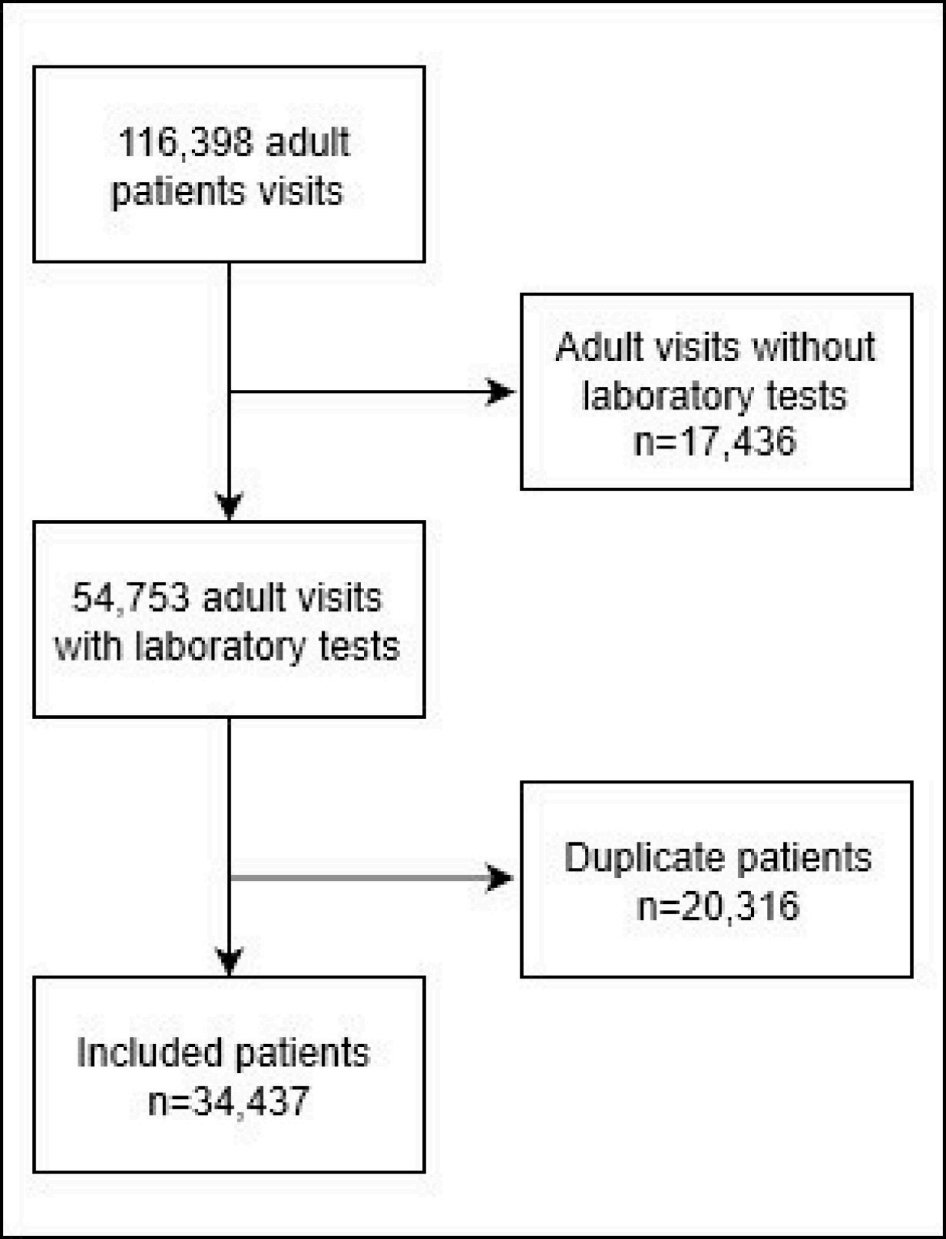


116,398 adult ED visits were recorded between January 2012 and December 2016. Laboratory testing was performed in 54,753 of these visits. Selecting only first ED visits, yielded 34,437 patients eligible for analysis (Fig 1). The majority of the population was male (54.8%). Median age (IQR) was 54 years (37–67). Admission rate was 55.7% and in total 1,942 (5.6%) patients died within 30 days after the ED visit. Patients who died presented to the ED with more abnormal vital signs (i.e. higher heart rate (93 vs. 87 per minute), lower systolic blood pressure (135 vs. 140 mmHg), abnormal consciousness level (37.3 vs. 7.5%), p<0.001) and were significantly older (68 vs. 52 years, p<0.001) (Table 2).

**Table 2:** Baseline characteristics.

	All patients	Died within 30-days	Alive	P-value
N (%)	34,437 (100%)	1,942 (5.6)	32,495 (94.4)	
**Demographics**				
*Male*, *N (%)*	18,827 (54.7)	1,187 (61.1)	17,640 (54.3)	<0.001
*Age*, *median (IQR)*	54 (37–67)	68 (58–78)	52 (36–66)	
**ED presentation characteristics**				
*Arrival by ambulance*, *N (%)*	10,387 (30.2)	1,074 (55.3)	9,313 (28.7)	<0.001
*Triage category*, *N (%)*^*a*^, immediate/very urgent	6,827 (19.8)	1,015 (52.3)	5,812 (17.9)	<0.001
**Vital signs**				
*Body temperature in °C*^*b*^, *mean (SD)*	36.8 (0.98)	36.2 (1.5)	36.9 (0.9)	<0.001
*Heart rate in bpm*^*c*^, *mean (SD)*	87 (21)	93 (25)	87 (21)	<0.001
*Systolic blood pressure in mmHg*^*d*^, *mean (SD)*	139 (27)	135 (39)	140 (26)	<0.001
*Diastolic blood pressure in mmHg*^*e*^, *mean (SD)*	82 (17)	77 (24)	82 (16)	<0.001
*Respiratory rate per minute*^*f*^, *mean (SD)*	19 (7)	21 (8)	19 (7)	<0.001
*Oxygen saturation in %*^*g*^, *median (IQR)*	98 (96–99)	97 (94–99)	98 (96–99)	<0.001
*Consciousness not alert*^*h*^, *N (%)*^*b*^	3,148 (9.1)	712 (37.3)	2,436 (7.5)	<0.001
**Admission, N (%)**	19,172 (55.7)	1,607 (84.2)	17,565 (54.0)	<0.001
**Laboratory tests**				
*Urea (mmol/L)*^*i*^, *mean (SD)*	6.7 (5.1)	9.7 (7.6)	6.5 (4.9)	<0.001
*Sodium (mmol/L)*^*j*^, *mean (SD)*	139 (4.4)	138 (6.1)	139 (4.2)	<0.001
*Glucose (mmol/L)*^*k*^, *mean (SD)*	7.4 (3.8)	8.9 (5.6)	7.2 (3.5)	<0.001
*Haemoglobin (g/dL)*^*l*^, *mean (SD)*	13.3 (2.1)	12.3 (2.6)	13.3 (2.1)	<0.001
*White blood cell count (10*^*9*^*/L)*^*m*^, *mean (SD)*	10.3 (18.3)	13.8 (14.6)	10.1 (18.4)	<0.001
*Platelets (10*^*9*^*/L)*^*n*^, *mean (SD)*	256 (104)	242 (133)	257 (243)	<0.001

Missing data are not yet imputed.

Abbreviations: °C, degrees Celsius; bpm, beats per minute; dL, decilitre; ED, emergency department; g, gram; IQR, interquartile range; L, litre; mmol, millimole; N, number; SD, standard deviation.

^a^Data on triage category were missing for 2,610 (7.6%) patients.

^b^Data on body temperature were missing for 10,995 (31.9%) patients.

^c^Data on heart rate were missing for 5,333 (15.5%) patients.

^d^Data on systolic blood pressure were missing for 5,499 (16.0%) patients.

^e^Data on diastolic blood pressure were missing for 5,452 (15.8%) patients.

^f^Data on respiratory rate were missing for 17,321 (50.3%) patients.

^g^Data on oxygen saturation were missing for 6,355 (18.5%) patients.

^h^Data on conscious level were missing for 9,837 (28.6%) patients.

^i^Data on urea level were missing for 9,640 (28.0%) patients.

^j^Data on sodium level were missing for 1,108 (3.2%) patients.

^k^Data on glucose level were missing for 955 (2.8%) patients.

^l^Data on haemoglobin level were missing for 421 (1.2%) patients.

^m^Data on white blood cell count were missing for 2,229 (6.5%) patients.

^n^Data on platelet count were missing for 1,786 (5.2%) patients.

### Model performance

Haemoglobin was most frequently tested (in 98.8% patients), whereas blood urea nitrogen was least often tested (72.0%). All predictive effects we found corresponded to the original model (e.g. low platelet count, haemoglobin level, sodium levels were associated with 30-day mortality) (Tables 1 and 2). Of all predictors in the model, age ≥ 65 years was the strongest predictor for 30-day mortality in univariate analysis (OR [95% CI] = 4.4 [4.0; 4.8]) (Table 3).

**Table 3:** Odds ratios [95% CI] for the full model and POCT model.

Parameter	Odds ratio	Odds ratio	Odds ratio
	[95% CI]	[95% CI]	[95% CI]
	*Univariate*	*Full model*	*POCT model*
Age ≥65 (years)	4.21 [3.83; 4.63]	2.73 [2.45; 3.05]	2.60 [2.34; 2.89]
Urea >7.0 (mmol/L)	3.24 [2.85; 3.67]	1.61 [1.39; 1.88]	1.71 [1.48; 1.98]
Haemoglobin <12.0 (g/dL)	2.57 [2.34; 2.83]	1.73 [1.56; 1.92]	1.82 [1.65; 2.02]
Sodium <135 (mmol/L)	2.63 [2.36; 2.96]	1.52 [1.35; 1.72]	1.64 [1.45; 1.85]
Glucose >7.0 (mmol/L)	4.36 [3.95; 4.81]	2.82 [2.54; 3.13]	3.15 [2.85; 3.50]
White blood cell count >10.0 (*10^9^/L)	2.39 [2.15; 2.66]	2.29 [2.05; 2.57]	NA
Platelet count <150 (*10^9^/L)	2.89 [2.57; 3.25]	2.79 [2.44; 3.19]	NA

Abbreviations: CI, confidence interval; dL, decilitre; g, gram; mmol, millimole; L, litre; NA, not applicable. The linear predictor of the full model can be calculated with the following formula: LP = -4.790 + 1.005*(Age ≥65) + 0.479*(Urea >7.0) + 0.548*(Haemoglobin <12.0) + 0.420*(Sodium <135) + 1.037*(Glucose >7.0) + 0.830*(White blood count >10.0) + 1.025*(Platelet count <150). The linear predictor of the POCT model: LP = -4.302 + 0.995*(Age ≥65) + 0.539*(Urea >7.0) + 0.601*(Haemoglobin <12.0) + 0.494*(Sodium <135) + 1.149*(Glucose >7.0). To determine the individual risk on 30-day mortality, apply the following formula: 1/(1 + exp(−linear predictor).

**Fig 2:**
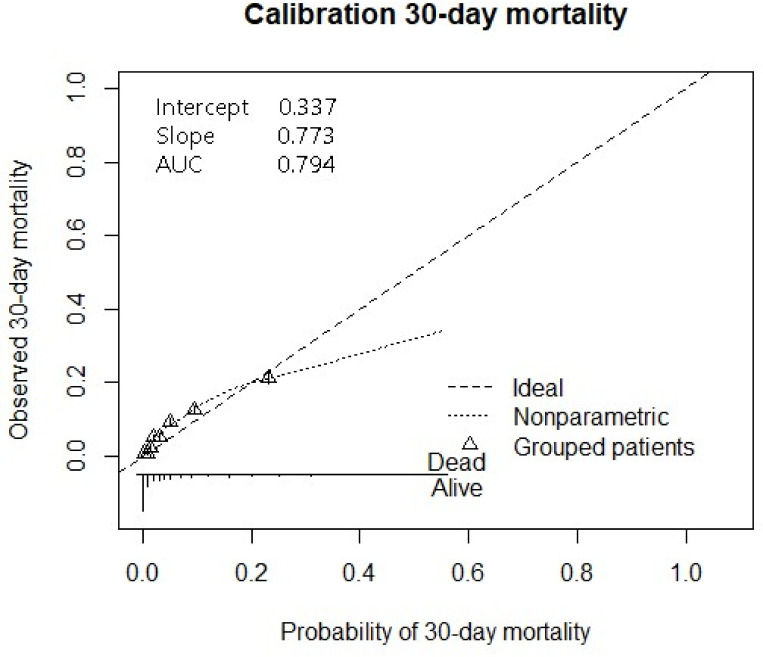


External validation showed an AUC of 0.796 [0.788–0.806] (S1 Fig). The calibration curve had a slope of 0.77 and an intercept of 0.34 (Fig 2).

We refitted the laboratory-based model on our own dataset with subsequent reduction to a model with only POCT parameters (Table 3). The refitted laboratory-based model yielded an internally validated AUC of 0.813, which slightly decreased to 0.790 when only including age and POCT parameters.

Likelihood ratios of score intervals of 0 to 5, 6 to 13 and 14 to 20 were 0.31, 1.77 and 5.04 respectively. Positive and negative likelihood ratios for dichotomous cut-off points are found in S1 Table. The highest Youden’s index was found using a cut-off point score of 8.

## Discussion

In this study we first externally validated the model by Asadollahi et al. that uses laboratory parameters and the patients’ age to predict mortality. Next we assessed that the Asadollahi et al. model based only on POCT available laboratory parameters yielded comparable performance. To our knowledge, we are the first to validate this laboratory-based model and to perform calibration. Our external validation resulted in a reasonable AUC of 0.796. One benefit of our study is that we used a large database for the external validation, which limits uncertainty in the performance of the model and thus increases the clinical relevance [18].

Despite the fact that most models are introduced without calibration, it is a critical step preceding implementation of a model. Calibration describes the agreement between the calculated, based on the prediction model, and the observed number of occurrences. The calibration of this model was suboptimal, which indicates the model slightly overestimates the mortality risk.

A major disadvantage of the study by Asadollahi et al. is the case-controlled study design. The authors included deceased and non-deceased patients in a 1:2 ratio, which yields a mortality rate of 33%. This results in an overestimation of the prevalence of 30-day mortality. Since we conducted a cohort study, our mortality rate reflects the 30-day mortality prevalence more accurately.

One of the merits of the model by Asadollahi et al. is that it uses only a few parameters. Additionally, the parameters within this model are virtually always assessed in patients with indication for laboratory diagnostics admitted to the ED. Therefore this model is generally applicable and easy to use by clinicians. Its simple interpretation accommodates usage implementation in electronic patient files. The power of laboratory values in prediction research is that they provide an objective measurement, especially compared to manually collected vital parameters. Vital parameters that are manually collected are prone to interrater variability. Also, vital parameters are subject to influences that are not always taken into account (pain, stress, normality for an individual patient). A downside of laboratory values is they take time to become available and are therefore difficult to implement in a decision model in the ED.

Ideally, prediction models in the ED should consist of readily available parameters and as little parameters as possible, making the model convenient for clinical practice. As most laboratory test results take more than an hour, the second aim of our study was to assess whether a model based on POCT parameters yielded similar predictive performance in predicting 30-day mortality. We found that our model with age and POCT parameters had similar performance. We provided the regression coefficients and an intercept allowing to replicate this study, but also to facilitate implementation of this model in clinical practice. POCT is promising since it only takes minutes to analyse blood samples. This may lead to a reduction in time to diagnosis and initiation of treatment. Furthermore, POCT-systems are already used in EDs. Although presently not every ED has a POCT-analyser, which may limit the applicability of this study, this study may encourage to invest in POCT-analysers.

A general limitation of literature concerning prediction models in the ED, is that hardly any study provides sufficient information to execute external validation. There are several models based on laboratory values published which performed well in general with an AUC above 0.80 [19–21].

A limitation of our study is its retrospective study design, which makes our study prone to bias. Nevertheless, laboratory data were automatically retrieved from the laboratory testing machines thus the quality of the data is high and not subject to human mistakes. Furthermore, we had missing data, mainly vital signs, which we replaced using multiple imputation. This is a valid way to manage even large samples of missing data [22], although a database with all data available is obviously superior. Therefore, we should strive to collect data as complete as possible. Last, this validation study took place in a tertiary care centre which corresponds to the derivation study [11]. Therefore, our results might be less generalizable to other centres with patients with different complexity and pathology. We therefore recommend external validation of our model in another centre, before implementation. In addition, we encourage considering POCT in prediction model development, researching both its discrimination and calibration.

In conclusion, the performance of the model by Asadollahi et al. was adequate in identifying patients at high risk for mortality in the ED. However, our POCT-model performs similar with results available within minutes. Although our model needs further validation and evaluation, it shows the potential of POCT in early risk stratification in the ED.

## Supporting information

S1 Fig AUC Lab model. (TIF)

S1 Table Sensitivity, specificity, LR+, LR-, Youden’s index for different cut-off points. (DOCX)

## Abbreviations

AUC: area under the curve
bpm: beats per minute
CI: confidence interval
dL: decilitre
ED: emergency department
Erasmus MC: Erasmus University Medical Center
ESI: Emergency Severity Index
EWS: Early Warning Scores
g: gram
IQR: interquartile range
L: litre
MEWS: Modified Early Warning Score
mmol: millimole
MTS: Manchester Triage Systems
NEWS: National Early Warning Score
POCT: point-of-care testing
ROC: Receiver Operating Characteristic
SD: standard deviation
