# Supplementary figures and images for: Predicting 30-day mortality using point-of-care testing; an external validation and derivation study
Source: PLoS One. 2020 Sep 24;15(9):e0239318. doi: 10.1371/journal.pone.0239318 (PMC7514068; doi:10.1371/journal.pone.0239318)

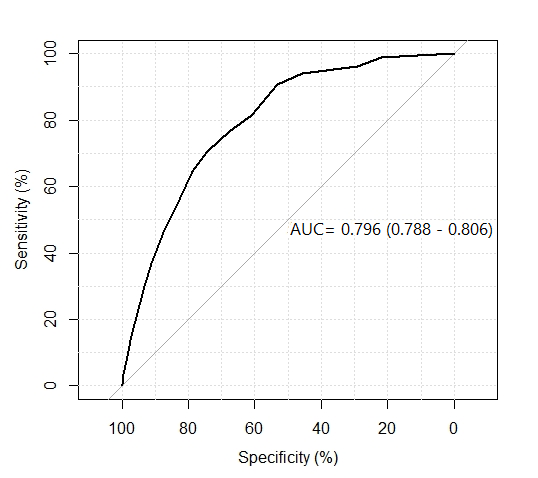

Supplement: S1 Fig — (TIF) [file pone.0239318.s001.tif]
