# Supplementary material for: Predicting 30-day mortality using point-of-care testing; an external validation and derivation study
Source: PLoS One. 2020 Sep 24;15(9):e0239318. doi: 10.1371/journal.pone.0239318 (PMC7514068; doi:10.1371/journal.pone.0239318)
Supplement: S1 Table — (DOCX) [file pone.0239318.s002.docx]

**S1 Table. Sensitivity, specificity, LR+, LR-, Youden’s index for different cut-off points**

| **Cut-off point** | **Sensitivity** | **Specificity** | **Positive likelihood ratio** | **Negative likelihood ratio** | **Youden's index** |
| --- | --- | --- | --- | --- | --- |
| 1 | 0,99 | 0,22 | 1,27 | 0,05 | 0,21 |
| 2 | 0,99 | 0,22 | 1,27 | 0,05 | 0,21 |
| 3 | 0,96 | 0,29 | 1,36 | 0,13 | 0,25 |
| 4 | 0,94 | 0,46 | 1,73 | 0,13 | 0,40 |
| 5 | 0,91 | 0,53 | 1,94 | 0,18 | 0,44 |
| 6 | 0,81 | 0,61 | 2,08 | 0,31 | 0,42 |
| 7 | 0,77 | 0,68 | 2,38 | 0,34 | 0,44 |
| 8 | 0,70 | 0,74 | 2,74 | 0,40 | 0,45 |
| 9 | 0,65 | 0,79 | 3,03 | 0,45 | 0,43 |
| 10 | 0,53 | 0,84 | 3,34 | 0,56 | 0,37 |
| 11 | 0,46 | 0,88 | 3,74 | 0,61 | 0,34 |
| 12 | 0,37 | 0,91 | 4,25 | 0,69 | 0,28 |
| 13 | 0,30 | 0,93 | 4,46 | 0,75 | 0,23 |
| 14 | 0,19 | 0,96 | 5,04 | 0,84 | 0,15 |
| 15 | 0,14 | 0,98 | 5,51 | 0,89 | 0,11 |
| 16 | 0,09 | 0,98 | 5,32 | 0,93 | 0,07 |
| 17 | 0,04 | 0,99 | 6,94 | 0,97 | 0,03 |
| 18 | 0,03 | 1,00 | 7,38 | 0,97 | 0,03 |
| 19 | 0,00 | 1,00 | 33,47 | 1,00 | 0,00 |
| 20 | 0,00 | 1,00 | NA | 1,00 | 0,00 |

Interval likelihood ratios for score 0 to 5, 6 to 13 and 14 to 20 were 0.31, 1.77 and 5.04. Positive likelihood ratio = sensitivity/(1-specificity). Negative likelihood ratio= (1-sensitivity)/specificity.
